# Endogenous Sulfur Dioxide Inhibits Vascular Calcification in Association with the TGF-β/Smad Signaling Pathway

**DOI:** 10.3390/ijms17030266

**Published:** 2016-02-23

**Authors:** Zhenzhen Li, Yaqian Huang, Junbao Du, Angie Dong Liu, Chaoshu Tang, Yongfen Qi, Hongfang Jin

**Affiliations:** 1 Department of Pediatrics, Peking University First Hospital, Beijing 100034, China; zhenzhenli1886@126.com (Z.L.); yaqianhuang@126.com (Y.H.); junbaodu1@126.com (J.D.); 2 Key Laboratory of Molecular Cardiology, Ministry of Education, Beijing 100191, China; tangchaoshu@263.net.cn; 3 Department of Medical and Health Sciences, Linköping University, Linköping 58183, Sweden; angie.dongliu@gmail.com; 4 Department of Physiology and Pathophysiology, Peking University Health Science Centre, Beijing 100191, China

**Keywords:** sulfur dioxide, vascular calcification, smooth muscle cell, transforming growth factor-β

## Abstract

The study was designed to investigate whether endogenous sulfur dioxide (SO_2_) plays a role in vascular calcification (VC) in rats and its possible mechanisms. *In vivo* medial vascular calcification was induced in rats by vitamin D3 and nicotine for four weeks. *In vitro* calcification of cultured A7r5 vascular smooth muscle cells (VSMCs) was induced by calcifying media containing 5 mmol/L CaCl_2_. Aortic smooth muscle (SM) α-actin, runt-related transcription factor 2 (Runx2), transforming growth factor-β (TGF-β) and Smad expression was measured. VC rats showed dispersed calcified nodules among the elastic fibers in calcified aorta with increased aortic calcium content and alkaline phosphatase (ALP) activity. SM α-actin was markedly decreased, but the osteochondrogenic marker Runx2 concomitantly increased and TGF-β/Smad signaling was activated, in association with the downregulated SO_2_/aspartate aminotransferase (AAT) pathway. However, SO_2_ supplementation successfully ameliorated vascular calcification, and increased SM α-actin expression, but inhibited Runx2 and TGF-β/Smad expression. In calcified A7r5 VSMCs, the endogenous SO_2_/AAT pathway was significantly downregulated. SO_2_ treatment reduced the calcium deposits, calcium content, ALP activity and Runx2 expression and downregulated the TGF-β/Smad pathway in A7r5 cells but increased SM α-actin expression. In brief, SO_2_ significantly ameliorated vascular calcification in association with downregulation of the TGF-β/Smad pathway.

## 1. Introduction

Vascular calcification (VC) is a common complication of aging, atherosclerosis, hypertension, diabetes and chronic kidney disease. It is closely associated with cardiovascular morbidity and mortality and is an important risk factor for many cardiovascular diseases [1,2]. Recently, studies have shown that vascular calcification is a cell-mediated and highly regulated process similar to osteogenesis [3]. And it is the key point that vascular smooth muscle cells (VSMCs) lose the expression of smooth-muscle lineage markers and express osteogenic markers [3].

As important vascular cells, VSMCs retain multi-potential capability. Under the influence of many factors, VSMCs can lose the expression of smooth-muscle lineage markers and transform into osteo-/chondrocytic-like cells, form matrix vesicle, and express the osteoblast related proteins. Then, the process of mineralization is activated [4]. Several molecules facilitate osteochondrogenic transition of VSMCs and vascular calcification, including Runx2/Cbfa1, bone morphogenetic protein (BMP), Msx2, osterix, and Sox9, *etc*. Recent studies have shown that microRNA regulates several key checkpoints in the cellular processes involved in the process of VSMC phenotype transformation [4,5,6,7]. These findings suggest that the mechanism for VSMC trans-differentiation during the pathogenesis of vascular calcification is a complex multifactorial and tightly regulated process, and possibly related to the regulation of genes, molecules, and signaling pathways. However, the underlying molecular and cellular mechanisms for vascular calcification are still not fully understood.

Endogenous sulfur dioxide (SO_2_) can be produced during the metabolism of sulfur-containing amino acids. Aspartate aminotransferase (AAT) is considered to be a key enzyme during SO_2_ generation [8]. SO_2_ has many physiological functions such as regulating blood pressure, vasorelaxation and regulation on cardiac function [8,9,10,11,12]. SO_2_ also plays an important role in many pathophysiological processes of various vascular diseases including hypertension and pulmonary arterial hypertension [13,14]. Previously, our research team found that SO_2_ had regulatory effects on vascular remodeling [10], via the inhibition of vascular VSMC proliferation [13]. We found that SO_2_ exerted a negative regulation of VSMC proliferation by suppressing the Erk/MAPK pathway mediated by cAMP/PKA signaling [15]. Consequently, we speculated that SO_2_ might participate in the regulation of vascular calcification process.

Therefore, in the present study, we used *in vivo* and *in vitro* experiments to investigate if SO_2_ was involved in the regulation of vascular calcification and the possible underlying mechanisms.

## 2. Results

### 2.1. Establishment of the Rat Vascular Calcification Rat Model

**Figure 1:**
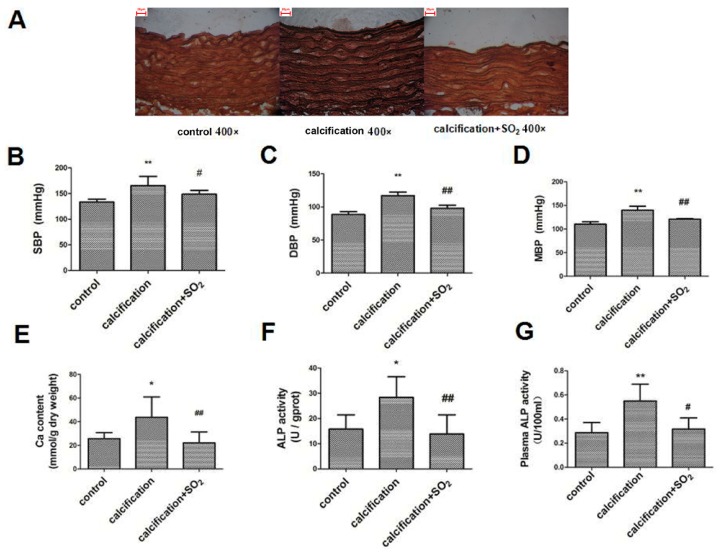
Sulfur dioxide (SO_2_) prevents vascular calcification *in vivo.* (**A**) Von Kossa staining of rat thoracic aorta. In the calcification group, there were dispersed calcified nodules among the elastic fibers. In the calcification + SO_2_ group, Von Kossa staining showed dispersed calcified nodules among the elastic fibers disappeared. Scale bar, 20 μm; (**B**–**D**) Measurement of hemodynamics; (**B**) systolic blood pressure (SBP); (**C**) diastolic blood pressure (DBP); (**D**) mean blood pressure (MBP); (**E**) Quantification of calcium content of rat thoracic aortic tissue; (**F**) Alkaline phosphatase (ALP) activity of rat thoracic aortic tissue; (**G**) Plasma ALP activity. ** *p* < 0.01 *vs.* control; * *p* < 0.05 *vs.* control; **#** *p* < 0.05 *vs.* calcification; **##** *p* < 0.01 *vs.* calcification (*n* = 10 per group).

Von Kossa staining showed dispersed calcified nodules among the elastic fibers in calcified aortas but not in control aortas (Figure 1A). Compared with control group, SBP, DBP and MBP in calcification group were significantly increased (*p* < 0.01, *p* < 0.01 and *p* < 0.01) (Figure 1B–D) and the aortic calcium content was markedly increased (*p* < 0.05) (Figure 1E). In addition, the alkaline phosphatase (ALP) activity in both aorta and plasma was increased (*p* < 0.01 and *p* < 0.05) (Figure 1F,G).

### 2.2. The SO_2_/AAT Pathway Is Downregulated in Calcified Arteries

**Figure 2:**
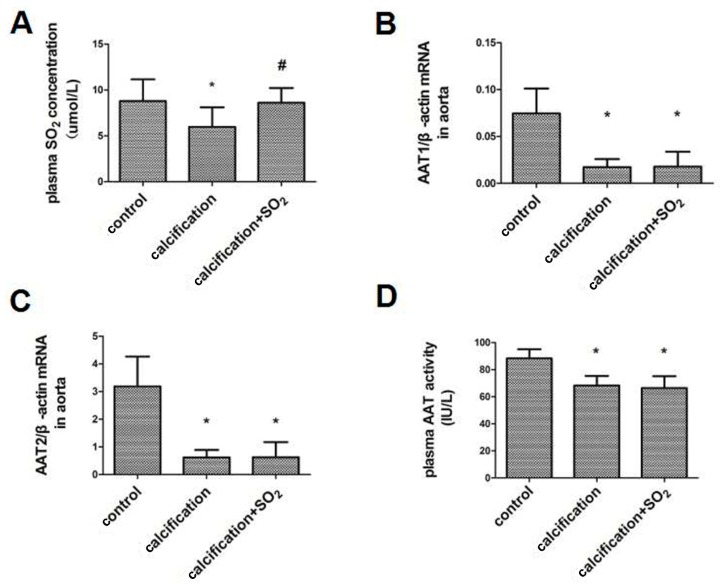
Endogenous SO_2_/aspartate aminotransferase (AAT) pathway in calcified arteries. (**A**) The plasma SO_2_ concentration; (**B**,**C**) The expression of AAT1 and AAT2 mRNA; (**D**) The plasma AAT activity. * *p* < 0.05 *vs.* control; **#** *p* < 0.01 *vs.* calcification (*n* = 10 per group).

The plasma SO_2_ content was lower in the calcification group than in control group (*p* < 0.05) (Figure 2A). RT-PCR analysis revealed a reduced expression of AAT1 and AAT2 mRNAs, key enzymes for SO_2_ production, in the calcified aortas compared to the controls (*p* < 0.05, *p* < 0.05) (Figure 2B,C). Moreover, AAT activity in the plasma was decreased in the calcification model (*p* < 0.05) (Figure 2D). Thus, the endogenous SO_2_/AAT pathway was downregulated in calcified arteries.

### 2.3. SO_2_ Treatment Attenuated Vascular Calcification in Vivo

After treatment with SO_2_, Von Kossa staining showed dispersed calcified nodules among the elastic fibers disappeared in the calcification + SO_2_ group as compared with the calcification group (Figure 1A). SBP, DBP and MBP were significantly decreased in rats of calcification + SO_2_ group (*p* < 0.05, *p* < 0.01 and *p* < 0.01, respectively) (Figure 1B–D). Furthermore, SO_2_ decreased the calcium content and ALP activity in both aorta homogenates and plasma (*p* < 0.01, *p* < 0.01 and *p* < 0.05, respectively) (Figure 1E–G).

### 2.4. SO_2_ Attenuates Osteoblastic Differentiation of Vascular Smooth Muscle Cells in Vivo

**Figure 3:**
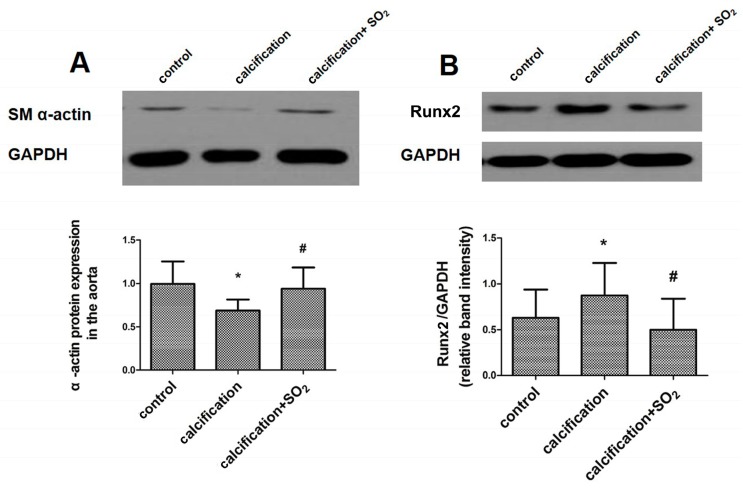
SO_2_ attenuates osteoblastic differentiation of vascular smooth muscle cells *in vivo.* Western blot analysis of SM α-actin (**A**) and Runx2 (**B**) in rat thoracic aortic tissue. * *p* < 0.05 *vs.* control; **#** *p* < 0.05 *vs.* calcification (*n* = 10 per group).

Compared with the normal vessels, the expression of the smooth muscle lineage marker SM α-actin was markedly decreased in aortas in the calcification group (*p* < 0.05, Figure 3A). In parallel, the level of the osteochondrogenic marker Runx2 concomitantly increased in aortas (*p* < 0.05, Figure 3B). However, SO_2_ treatment significantly prevented the upregulation of Runx2 and circumvented the reduction in the SM α-actin level (*p* < 0.05, *p* < 0.05, Figure 3A,B).

### 2.5. SO_2_ Inhibits Activation of the TGF-β Signaling Pathway during the Vascular Calcification Process

**Figure 4:**
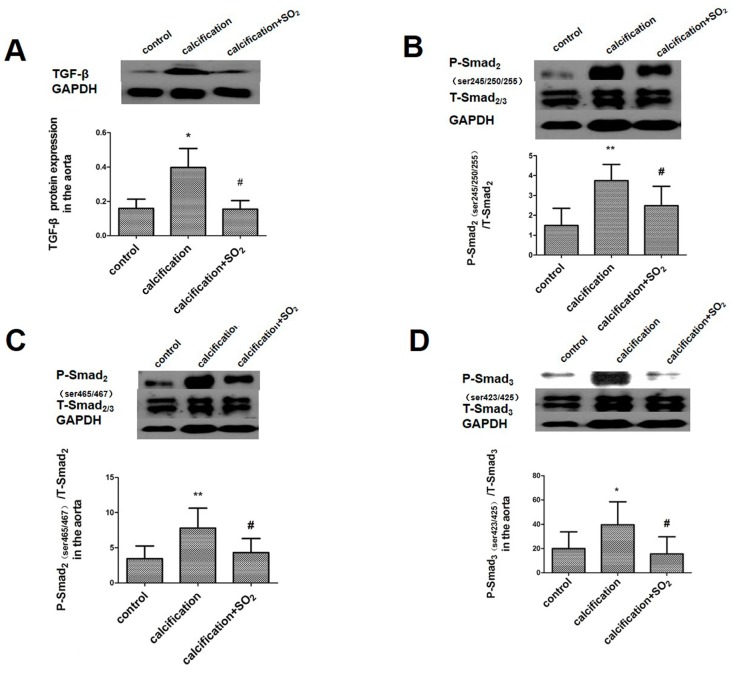
SO_2_ inhibits the activation of TGF-β signaling pathway during the process of vascular calcification *in vivo.* Western blot analysis of TGF-β (**A**); phospho-Smad2 (Ser245/250/255) (**B**); phospho-Smad2 (Ser465/467) (**C**); and phospho-Smad3 (Ser423/425) (**D**) in rat thoracic aortic tissue. * *p* < 0.05 *vs.* control; ** *p* < 0.01 *vs.* control; **#** *p* < 0.05 *vs.* calcification (*n* = 10 per group).

Compared with the control group, the expressions of TGF-β and its downstream signaling molecules P-Smad2 (ser245/250/255), P-Smad2 (ser465/467) and P-Smad3 (ser423/425) in the aortic homogenate were increased significantly (*p* < 0.05, *p* < 0.01, *p* < 0.01 and *p* < 0.05, Figure 4). After treatment with SO_2_, however, the expression of these proteins was markedly decreased in rats of calcification group (*p* all< 0.05, Figure 4A–D). 

### 2.6. Establishment of the VSMC Calcification Model in Vitro

**Figure 5:**
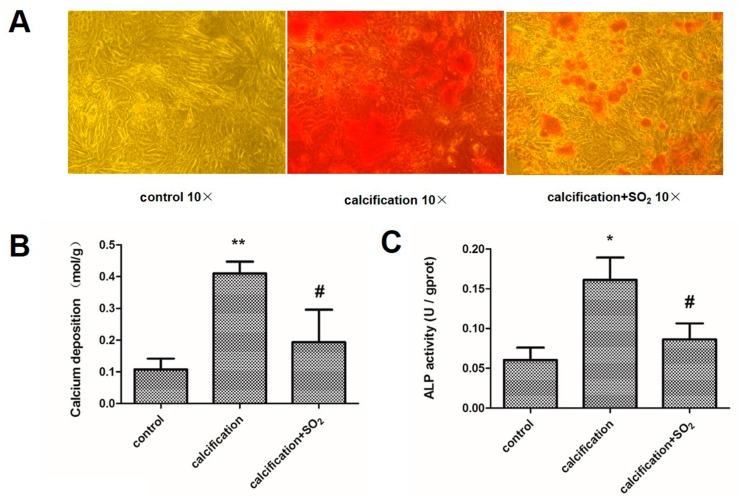
SO_2_ prevents vascular smooth muscle cell calcification *in vitro*. (**A**) Alizarin Red S staining in A7r5 vascular smooth muscle cells (VSMCs); (**B**) Quantification of calcium content in A7r5 VSMCs; (**C**) ALP activity assay in A7r5 VSMCs. ** *p* < 0.01 *vs.* control; * *p* < 0.05 *vs.* control; **#** *p* < 0.05 *vs.* calcification. Data are from three independent experiments (*n* = 3) performed in triplicate.

To further verify the above observation, we induced mineralization in incubated A7r5 cells in calcification media containing 5 mmol/L CaCl_2_. Compared with the control group, the VSMCs maintained in calcification medium showed development of granular calcium deposits throughout the cell culture (Figure 5A). Moreover, the calcium content and ALP activity of the cells were increased significantly (*p* < 0.01, *p* < 0.05, Figure 5B,C).

### 2.7. The Expression of the SO_2_/AAT Pathway in Calcified VSMCs

**Figure 6:**
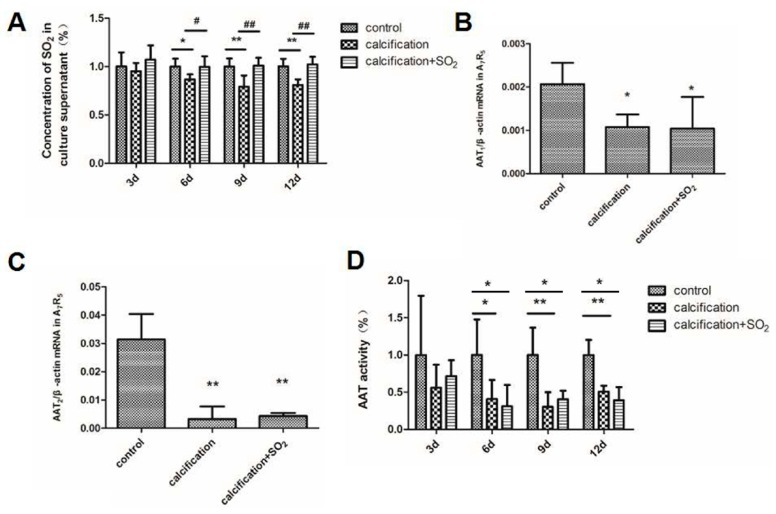
Change of endogenous SO_2_/AAT pathway in calcification VSMCs *in vitro*. (**A**) The SO_2_ concentration in A7r5 cell supernatants; (**B**,**C**) The expression of AAT1and AAT2 mRNA in A7r5 VSMCs; (**D**) The AAT activity in A7r5 cell supernatant. * *p* < 0.05 *vs.* control; ** *p* < 0.01 *vs.* control; **#** *p* < 0.05 *vs.* calcification; **##** *p* < 0.01 *vs.* calcification. Data are from three independent experiments (*n* = 3) performed in triplicate.

On the 3rd day, there were no differences in the culture supernatant SO_2_ content between the control group and the calcification group (*p* > 0.05). But on the 6th, 9th and 12th day the culture supernatant SO_2_ content in the calcification group was significantly reduced compared with that in the control group (*p* < 0.05, *p* < 0.01 and *p* < 0.01, Figure 6A). In the calcified cells, the expression of AAT1 and AAT2 mRNAs were significantly reduced (*p* < 0.05, *p* < 0.01, Figure 6B,C). Compared with the control group, the AAT activity did not alter after 3 days of the incubation with calcification media containing 5 mmol/L CaCl_2_ (*p* < 0.05), but on the 6th, 9th or 12th day the AAT activity was significantly decreased (*p* < 0.05, *p* < 0.01 and *p* < 0.01, Figure 6D). 

### 2.8. SO_2_ Treatment Attenuates Cell Calcification in Vitro

Compared with the calcification group, Alizarin red S staining showed the granular calcium deposits disappeared after treatment with SO_2_ (Figure 5A). With treatment of SO_2_, the calcium content and ALP activity were lower than those in the calcified VSMCs (*p* < 0.05, *p* < 0.05, Figure 5B,C).

### 2.9. SO_2_ Inhibits Osteoblastic Differentiation of VSMCs in Vitro

**Figure 7:**
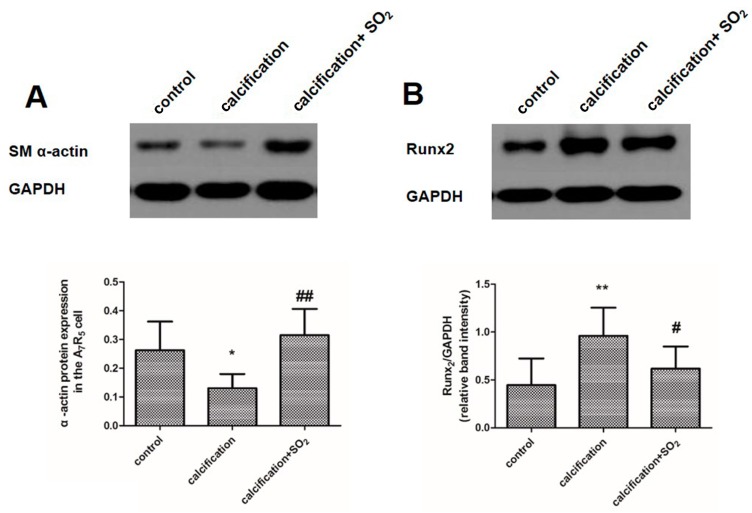
SO_2_ inhibits osteoblastic differentiation of vascular smooth muscle cells *in vitro*. Western blot analysis of SM α-actin (**A**) and Runx2 (**B**) in A7r5 VSMCs. * *p* < 0.05 *vs.* control; ** *p* < 0.01 *vs.* control; **#** *p* < 0.05 *vs.* calcification; **##** *p* < 0.01 *vs.* calcification. Data are from three independent experiments (*n* = 3) performed in triplicate.

In the calcified cells, the expression of SM α-actin was significantly decreased, while the level of osteochondrogenic marker Runx2 was markedly increased (*p* < 0.05, *p* < 0.01, Figure 7A,B). But SO_2_ treatment significantly prevented the upregulation of Runx2 and circumvented the reduction in SM α-actin level (*p* < 0.01, *p* < 0.05, Figure 7A,B).

### 2.10. SO_2_ Inhibits Activation of the TGF-β Signaling Pathway during the Process of VSMCs Calcification 

**Figure 8:**
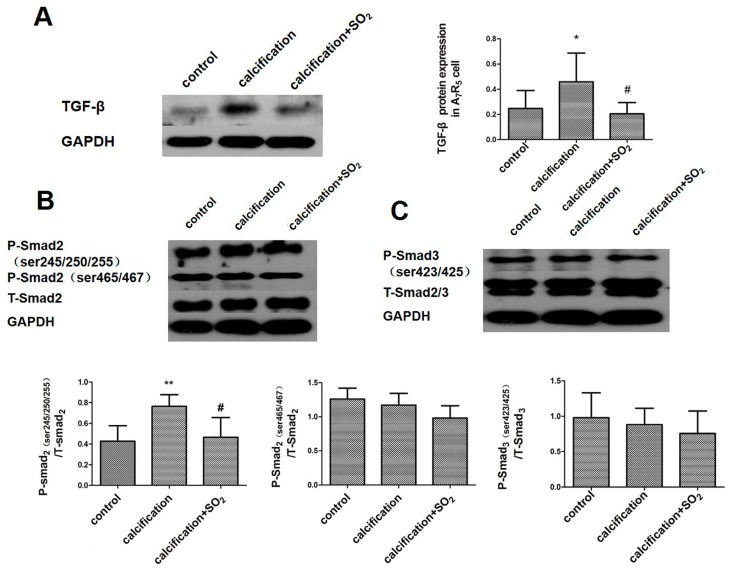
SO_2_ inhibited the activation of TGF-β signaling pathway during the process of VSMCs calcification *in vitro*. Western blot analysis of TGF-β (**A**) and Smads (**B**,**C**) in A7r5 VSMCs. * *p* < 0.05 *vs.* control; ** *p* < 0.01 *vs.* control; # *p* < 0.05 *vs.* calcification. Data are from three independent experiments (*n* = 3) performed in triplicate.

The expression of TGF-β (Figure 8A) and its downstream signaling molecules P-Smad2 (ser245/250/255) in the calcified cells was increased significantly (*p* < 0.01, Figure 4D), but the P-Smad2 (ser465/467) and P-Smad3 (ser423/425) expression had no significant differences compared with the control group (*p* < 0.05, *p* < 0.05, Figure 8B,C). With treatment of SO_2_, however, the expression of TGF-β and P-Smad2 (ser245/250/255) was decreased significantly (*p* < 0.05, *p* < 0.05, Figure 8A,B) but the P-Smad2 (ser465/467) and P-Smad3 (ser423/425) expression did not change in the calcification group (*p* all > 0.05, Figure 8B,C).

## 3. Discussion

More and more studies have shown that vascular calcification is a cell-mediated, active, and highly regulated process. This process may be associated with many mechanisms including the disorder of calcium-phosphorus metabolism, osteoblast phenotype transformation of vascular smooth muscle cells and imbalance of promoter/inhibitors of vascular calcification [3]. But the exact mechanisms are still unclear. SO_2_ is a new endogenous gaseous transmitter [8]. Our group found that SO_2_ could be produced endogenously in the cardiovascular system and we showed the presence of the key enzyme AAT in various organs and tissues of rats [8]. We also found that SO_2_ has marked cardiovascular, physiological and pathophysiological significance [8,9,10,11,12,13,14,16,17,18,19,20].

In our study, we established the calcification model *in vivo* and *in vitro,* and investigated the endogenous SO_2_/AAT pathway. We found that the expression of the endogenous SO_2_/AAT pathway was downregulated in the calcification model both *in vivo* and *in vitro*. Interestingly, SO_2_ supplementation could significantly increase the plasma level of SO_2_ and inhibit vascular calcification. Meanwhile, SO_2_ suppressed VSMC calcification, exhibiting loss of granular calcium deposits in calcified VSMCs and decreased calcium content and ALP activity. These results suggest that the downregulated endogenous SO_2_/AAT pathway is involved in the pathogenesis of vascular calcification. Since AAT is a key enzyme during SO_2_ generation, AAT level did not change after the addition of SO_2_ derivatives, as we expected. This is because SO_2_ as a product by the enzyme AAT would not necessarily induce a significant change in the protein level of AAT itself [13].

The osteoblast transformation of VSMCs was known as a key process of calcification. In the process of calcification, VSMCs can lose the expression of smooth-muscle lineage markers, acquire osteoblast-like phenotype and express osteogenic markers [2,4]. We found that the expression of the smooth-muscle lineage marker SM α-actin was markedly decreased, whereas the level of the osteochondrogenic marker Runx2 was concomitantly increased in calcified aortas and cells. However, SO_2_ treatment significantly prevented the upregulation of Runx2 and circumvented the reduction in SM α-actin level. These results indicated that SO_2_ could inhibit the trans-differentiation of VSMCs. Collectively, the results demonstrated that bone mineralization and osteoblast transformation of VSMCs were present during the calcification process in association with the downregulation of the SO_2_/AAT pathway. Interestingly, we found that SO_2_ could prevent the bone mineralization and the development of an osteogenic phenotype in VSMCs. These findings suggested that SO_2_ played an important role in vascular calcification likely by inhibiting the bone mineralization and osteoblast transformation of VSMCs.

Then, we further explored the possible mechanism by which SO_2_ inhibited vascular calcification. TGF-β regulates cell proliferation, differentiation and apoptosis by autocrine or paracrine signaling via cell surface receptor signal transduction pathways and plays an important role in regulating extracellular matrix synthesis. Studies have shown that TGF-β1 exists in calcified aortic valve and can regulate interstitial cell calcification of aortic valve via an apoptosis mechanism [21,22]. Other studies have shown that TGF-β can regulate vascular calcification and the differentiation of VSMCs *in vivo* [23,24]. TGF-β transmits cytoplasmic signals into the intracellular domain via its type I and II receptors. The activated type II receptor combines with the type I receptor to form a heterotrimer which consists of type II receptor-ligand-type I receptors. The activated type II receptor phosphorylates Smad2 and Smad3 (receptor-regulated Smad proteins, R-Smad) and transmits cytoplasmic signals into intracellular signals. The activated phosphorylated Smad2 and Smad3 combine with Smad4 (common mediated Smad, co-Smad) and translocate into the nucleus to regulate the transcription of many genes [25]. In our study, we found that the expression of TGF-β was increased and meanwhile the phosphorylation of its downstream signal molecules Smad2/3 was reinforced in calcified vascular tissues and cells. It was suggested that the activation of the TGF-β signal pathway was enhanced in the process of vascular calcification. We also found that the increased expression of TGF-β and phosphorylation of its downstream signal molecules were inhibited after treatment with SO_2_. Studies have shown that Runx2 is a key regulatory factor for vascular calcification and Runx2/Cbfa1 is a key regulatory factor for the phenotypic transformation of smooth muscle cells and matrix calcification under the hyperphosphate condition. The upregulation of the Runx2/Cbfa1 expression is crucial for the osteogenesis/ chondrogenesis phenotypic transformation and the start of the process of calcification, which was seen in the process of vascular calcification in patients with chronic kidney disease [26]. Another study showed that Runx2, as a common and important target gene of the TGF-β and BMP signal pathway, together with receptor-activated Smad protein, could stop C2C12 cell myogenic differentiation and induce its osteoblast differentiation [27]. In our study, we found that the expression of TGF-β was increased and its phosphorylation of downstream signal molecules was reinforced together with the upregulation of Runx2 expression in the calcified vascular tissues and cells. However, SO_2_ inhibited the upregulation of the expression of TGF-β and the enhancement of its downstream signal molecule phosphorylation, and the expression of Runx2 was reduced. These findings suggest that the TGF-β signal pathway is probably involved in the inhibition of vascular calcification by SO_2_.

However, the present study has limitations. It is known that vascular calcification is related to arterial hypertension and to the decreased vascular compliance, with subsequent elevated systolic blood pressure [28]. Several studies showed that administration of vitamin D_3_ plus nicotine could induce severe medial calcification of the aorta and produce a significant increase in blood pressure of rats [29,30]. Our present study demonstrated that SO_2_ treatment markedly attenuated vascular calcification and decreased blood pressure in rats. However, the attenuation in blood pressure by SO_2_ might be related not only to the inhibition of vascular calcification, but also to the vasorelaxation induced by SO_2_ [8]. Also, previous studies indicated that the multi-organ calcification including cardiac and renal calcification could be induced by vitamin D_3_ plus nicotine [30]. Therefore, we could not exclude the possibility that attenuation of blood pressure might also be associated with the inhibitory effect of SO_2_ on the cardiac and renal calcification and these issues need to be further elucidated. In addition, in the present study we measured arterial blood pressure under anaesthesia. It is known that the blood pressure is affected by anaesthetic depth although the control group was used to ensure the equivalent levels of anaesthesia in the present study.

## 4. Materials and Methods

### 4.1. Preparation of the Animal Model

All animal care and experimental protocols were performed in accordance with the Guide to The Care and Use of Experimental Animals issued by the Ministry of Health of the People’s Republic of China. Healthy male Sprague-Dawley rats (180–200 g) were provided by the Animal Research Center of Peking University First Hospital. All animals used in this work received humane care in compliance with the animal care guidelines of the institute and the study was approved by the Experimental Animal Ethic Committee of Peking University First Hospital. Thirty rats were randomly divided into three groups: control group (*n* = 10), calcification group (*n* = 10) and SO_2_ treatment (calcification + SO_2_) group (*n* = 10). Rats in calcification group and SO_2_ treatment group were subjected to vitamin D_3_ (300,000 IU /kg in arachis oil, intramuscularly) plus nicotine (25 mg/kg in 5 mL peanut oil, orally) (Sigma, St. Louis, MO, USA) at 9 am on day 1 and nicotine was re-administered at 7 pm [31]. Starting from second day, rats of calcification + SO_2_ group were given intraperitoneal injection of SO_2_ donor for 28 days. Na_2_SO_3_/NaHSO_3_ was freshly dissolved in physiological (0.9%) saline at 0.54 mmol/kg:0.18 mmol/kg before injection. We injected rats in the calcification and control groups with the same volume of physiological saline.

### 4.2. A7r5 VSMC Culture and Calcification Inducement

Rat A7r5 VSMCs were purchased from the American Type Culture Collection (Manassas, VA, USA). A7r5 cells (5 × 10^3^) were seeded in 6-well plates and the next day we started our treatment for 12 days. Calcification of A7r5 VSMCs was induced by calcifying media containing 5 mmol/L CaCl_2_ for 12 days with media changes every 2 days [2]. Smooth muscle cells in calcification + SO_2_ group were treated with 50 μmol/L SO_2_ with media changes every 2 days.

### 4.3. Measurement of Hemodynamic Features in Vivo

After 28 days treatment, all rats were anesthetized intraperitoneally with sodium pentobarbital (45 mg/kg). A silicone catheter filled with heparin saline (500 U/mL) was inserted into the right carotid arteries to measure systolic blood pressure (SBP), diastolic blood pressure (DBP) and mean blood pressure (MBP). The catheter was connected to a Multi-Lead Physiological Monitor (BL-420F, Chengdu TME Technology, Chengdu, China). The anaesthesia and the surgery conducted were blinded to the group identity of the rats.

### 4.4. Sample Preparation

Plasma was collected from the rats, centrifuged, and stored at −80 °C. Rat aorta was rapidly isolated. A consistent location of thoracic aorta from each rat was fixed in 4% polyoxymethylene for morphometric analysis. The rest of the aorta was frozen and stored in liquid nitrogen.

### 4.5. Quantification of Calcium Content

Calcium content in the aorta was measured as described [29]. The aortas were dried at 55 °C and weighed. Then dissolved in HNO_3_, dried in an oven at 180 °C and re-dissolved with the blank solution (27 nmol/L KCl and 27 μmol/L LaCl_3_ in deionized water). The calcium levels were determined by colorimetry through a reaction with the o-cresolphthalein complexon method (Calcium Kit, BioSino Bio-technology and Science Inc., Beijing, China) and were normalized by aortic dry weight.

For calcium content in the A7r5 VSMCs [2], A7r5 VSMCs were grown in 6-well plates and treated with growth medium or calcifying medium for 12 days. After removing the culture medium, cells were washed with phosphate buffered saline (PBS) and treated with 0.6 N HCl overnight at 4 °C. Then, the HCL supernatant was harvested and the total protein of cells was extracted. The calcium content in the HCL supernatant was determined by a calcium kit and was normalized by protein concentration.

### 4.6. ALP Activity Assay

Plasma and aortic tissue homogenate ALP activity was measured colorimetrically as the hydrolysis of p-nitrophenyl phosphate by ALP assay kit (Jiancheng Bioengineering Institute, Nanjing, China). ALP activity was measured and the results were normalized to levels of total protein.

### 4.7. Von Kossa Staining and Alizarin Red S Staining

Von Kossa staining of thoracic aortic ring calcification was performed as described previously [16]. A consistent location of thoracic aorta was used for all rats [32]. And the Alizarin red staining of A7r5 VSMCs was carried out in accordance with the predecessors [2].

### 4.8. Real-Time PCR Analysis

Total RNA from aortic tissue or A7r5 VSMCs was isolated with the use of TRIZOL reagent (Invitrogen, Carlsbad, CA, USA) and reverse-transcribed into single-strand cDNA using oligo (dT) 15 primer and M-MLV reverse transcriptase. Quantitative real-time polymerase chain reaction (RT-PCR) was performed with an ABI 7300 real-time PCR system (Applied Biosystems, Foster, CA, USA) and the amplification conditions were 95 °C for 5 min, followed by 40 cycles of 95 °C for 15 s and 60 °C for 1 min. Primers for rat AAT_1_ were: 5′-CCAGGGAGCTCGGATCGT-3′ (sense), and 5′-GCCATTGTCTTCACGTTTCCTT-3′ (antisense); primers for rat AAT_2_ were 5′-GAGGGTCGGAGCCAGCTT-3′ (sense), and 5′-TTTCCCCAGGATGGTTTGG-3′ (antisense); and primers for rat β-actin were 5′-ACCCGCGAGTACAACCTTCTT-3′ (sense) and 5′-TATCGTCATCCATGGCGAACT-3′ (antisense).

### 4.9. Western Blot Analysis

Extracts containing equal amounts of total protein from rat arteries or A7r5 VSMCs were resolved by SDS-PAGE and then transferred to a nitrocellulose membrane. The membranes were incubated with primary antibody and secondary antibody. The immunofluorescence signal was exposed to X-ray film (Kodak Scientific, New Haven, CT, USA), and then quantified by using AlphaImager (San Leandro, CA, USA).

### 4.10. Determination of SO_2_ Content in Plasma and Cell Supernatant

Samples obtained from plasma and cell supernatant were prepared and the high-performance liquid chromatography (HPLC, Agilent 1200 series, Agilent Technologies, Santa Clara, CA, USA) with fluorescence determination to determine the SO_2_ concentration [13,14].

### 4.11. AAT Activity Assay

AST/GOT Determination Kit (Nanjing Jiancheng Biological Engineer Academy, Nanjing, China) was used to detect AAT activity in plasma and cell supernatant. The samples were prepared according to the introduction.

### 4.12. Statistical Analysis

Results are expressed as mean ± S.D. Statistical comparisons were performed with SPSS 20.0 software (SPSS, Chicago, IL, USA). Comparisons between more than two groups involved one-way ANOVA followed by LSD test, and *p* < 0.05 was considered statistically significant. 

## 5. Conclusions

In our study, we observed a downregulation of the endogenous SO_2_/AAT pathway in the process of vascular calcification and the downregulated SO_2_/AAT pathway was likely involved in the development of vascular calcification. Mechanistically, we suggested that SO_2_ inhibited vascular calcification, likely in association with inhibition of the TGF-β signal pathway. These findings are meaningful for understanding the mechanisms for vascular calcification and also to provide new thoughts and potential strategies for the prevention and therapy of vascular calcification.
